# Phenotypic plasticity of fungal traits in response to moisture and temperature

**DOI:** 10.1038/s43705-021-00045-9

**Published:** 2021-08-28

**Authors:** Charlotte J. Alster, Steven D. Allison, Nels G. Johnson, Sydney I. Glassman, Kathleen K. Treseder

**Affiliations:** 1 Ecology and Evolutionary Biology, University of California Irvine, Irvine, CA USA; 2 School of Science, University of Waikato, Hamilton, New Zealand; 3 Department of Earth System Science, University of California, Irvine, Irvine, CA USA; 4 USDA Forest Service, Pacific Southwest Research Station, Albany, CA USA; 5 Department of Microbiology and Plant Pathology, University of California, Riverside, CA USA

**Keywords:** Microbial ecology, Microbial ecology, Fungal ecology, Climate-change ecology

## Abstract

Phenotypic plasticity of traits is commonly measured in plants to improve understanding of organismal and ecosystem responses to climate change but is far less studied for microbes. Specifically, decomposer fungi are thought to display high levels of phenotypic plasticity and their functions have important implications for ecosystem dynamics. Assessing the phenotypic plasticity of fungal traits may therefore be important for predicting fungal community response to climate change. Here, we assess the phenotypic plasticity of 15 fungal isolates (12 species) from a Southern California grassland. Fungi were incubated on litter at five moisture levels (ranging from 4–50% water holding capacity) and at five temperatures (ranging from 4–36 °C). After incubation, fungal biomass and activities of four extracellular enzymes (cellobiohydrolase (CBH), *β*-glucosidase (BG), *β*-xylosidase (BX), and N-acetyl-*β*-*D*-glucosaminidase (NAG)) were measured. We used response surface methodology to determine how fungal phenotypic plasticity differs across the moisture-temperature gradient. We hypothesized that fungal biomass and extracellular enzyme activities would vary with moisture and temperature and that the shape of the response surface would vary between fungal isolates. We further hypothesized that more closely related fungi would show more similar response surfaces across the moisture-temperature gradient. In support of our hypotheses, we found that plasticity differed between fungi along the temperature gradient for fungal biomass and for all the extracellular enzyme activities. Plasticity also differed between fungi along the moisture gradient for BG activity. These differences appear to be caused by variation mainly at the moisture and temperature extremes. We also found that more closely related fungi had more similar extracellular enzymes activities at the highest temperature. Altogether, this evidence suggests that with global warming, fungal biodiversity may become increasingly important as functional traits tend to diverge along phylogenetic lines at higher temperatures.

## Introduction

Few trait-based approaches in soil microbial ecology assess phenotypic plasticity as a trait [1, 2]. Organisms with high phenotypic plasticity exhibit a wide range of physiological or morphological changes in response to different environmental conditions [3]. Phenotypic plasticity is commonly studied in plants to improve understanding of organismal and ecosystem responses to climate change [4–7], yet far less studied in microbes [2, 8]. Phenotypic plasticity can influence the extent and speed with which microbial communities respond to climate change, independent from shifts in community composition or evolutionary adaptation [5]. Thus, the phenotypic plasticity of microbes could be a critical element in ecosystem models [9].

Microbes are thought to exhibit a high degree of phenotypic plasticity in order to reduce environmental threats to growth and propagation [10–12], with consequences for biogeochemical cycling [13, 14]. Decomposer fungi, for example, play a critical role in the global carbon (C) and nitrogen (N) cycling [15]. If these fungi have high phenotypic plasticity, they could quickly alter ecosystem dynamics by changing soil properties and rates of nutrient cycling [16, 17]. For example, fungal individuals experiencing moisture stress could decrease extracellular enzyme production, which would slow rates of decomposition in ecosystems [18, 19]. Or, under optimal temperature conditions, fungi could expand their hyphal networks [20, 21], resulting in improved aggregate stability and water retention in soils [22–24]. Therefore, phenotypic plasticity is important to consider when predicting organismal responses to climate change [2, 25].

Soil fungi may be particularly phenotypically plastic. For example, dimorphic yeasts can switch morphologies between single-celled yeast and multicellular hyphal structures, depending on the environment [15, 26]. In addition, soil fungi can be resistant to disturbance (e.g., drought and warming), which can indicate high phenotypic plasticity [27, 28]. Yet, microbial phenotypic plasticity studies tend to focus on bacteria and aquatic environments, rather than fungi in soils [29, 30].

A relatively large body of evidence has documented that fungal communities or populations respond to temperature and moisture [1, 14, 30–33], especially with respect to their extracellular enzyme activity (EEA) and biomass (e.g., [34, 35]). These changes could result from phenotypic plasticity, community shifts, or evolution. Another study isolated the effects of phenotypic plasticity by focusing specifically on individual fungal isolates [36]. In particular, they found that several strains of *﻿Neurospora discreta* (phylum Ascomycota) varied in the degree to which potential activities of extracellular enzymes changed with temperature. In another individual-based study, growth rates of two strains of ﻿*Trichoderma virens* (phylum Ascomycota) varied similarly with temperature, indicating that they did not differ appreciably in phenotypic plasticity [37]. If we understand the extent to which phenotypic plasticity varies among fungal individuals within a given community, we can assess the value of incorporating phenotypic plasticity as a trait in ecosystem models.

Microbial traits can also vary phylogenetically [38–41]. More closely related taxa may show more similar trait values if those traits are phylogenetically conserved, meaning that taxa with greater genetic relatedness would share more similar trait values [39]. Knowledge of phylogenetic distributions of fungal traits could be useful in predicting traits of unstudied organisms and linking microbial community composition to ecosystem models [41]. For example, if phenotypic plasticity is phylogenetically related, then we can infer the phenotypic plasticity of a given fungal taxon based on the phenotypic plasticity of its relatives.

To examine phenotypic plasticity in fungi under simulated climate change, we incubated isolates from grassland litter under a range of temperature and moisture conditions and measured their potential EEA and biomass production. We focused on potential EEA and fungal biomass because they are sensitive to changes in temperature and moisture [42–44] and have implications for biogeochemical cycling [45, 46]. Here, we refer to a fungal isolate’s variation in potential EEA and biomass across a temperature-moisture gradient as its *plasticity trait*. For example, the plasticity trait of fungal biomass would be the topography, or response surface, of all the biomass values across the temperature-moisture gradient. In addition, we define the term *point value* as the EEA or biomass measured at a specific temperature and moisture level for a given isolate. The point value of fungal biomass would be the biomass measured at a given temperature and moisture (e.g., at 20 °C and 50% moisture).

**Fig. 1:**
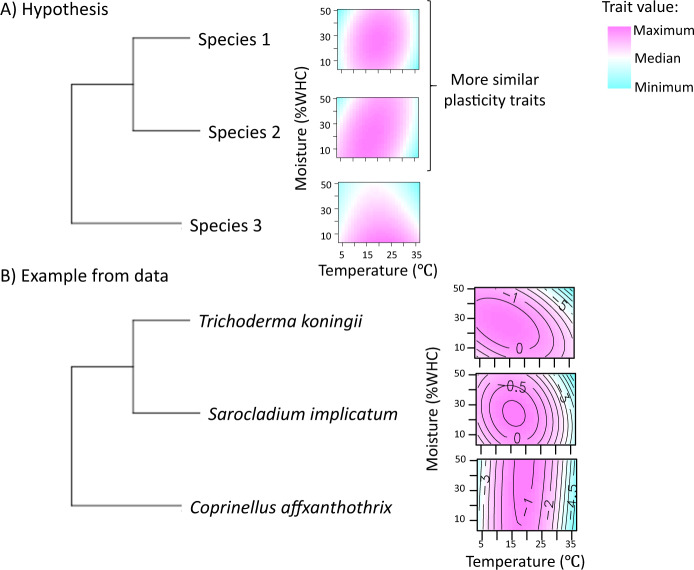
Conceptual figure of hypotheses and example. **A** Conceptual figure demonstrating traits as a function of both environmental (temperature and moisture) and genetic properties. Temperature and moisture are independent variables and the trait values are the dependent variables. Maximum trait values are in pink and minimum values are blue. We predicted that across fungal isolates, point values (i.e., enzyme activity or biomass at a given temperature and moisture level) should vary with temperature and moisture (Hypothesis 1). In addition, plasticity traits (i.e., enzyme responses or biomass across the temperature-moisture gradient) should vary between fungal isolates (Hypothesis 2). We also predicted that more similar fungi should exhibit more similar point values at specific points along with the moisture and temperature gradient (Hypothesis 3). **B** Examples of three response surfaces for N-acetyl-*β*-*D*-glucosaminidase (NAG). The numbers on the plot (panel **B**) represent the values of the contour lines (values are logged). In this example, the point values from the three different isolates are similar in the center of the response surface. However, moving to the edges of the temperature gradient, NAG activity for *Coprinellus aff xanthotrhis* (phylum Basidiomycota) diverges from *Trichoderma koningii* (phylum Ascomycota) and *Sarocladium implicatum* (phylum Ascomycota), the more phylogenetically similar species. The phylogeny for all of the fungi used in this experiment is in Figure S1.

We tested three hypotheses (Fig. 1A). First, across fungal isolates, point values of traits should vary with moisture and temperature (Hypothesis 1). Second, plasticity traits should differ among fungal isolates (Hypothesis 2). Third, more closely related fungi will show more similar point values at any given moisture and temperature point across the moisture-temperature gradient (Hypothesis 3).

## Methods

### Experimental design

To measure fungal plasticity traits and point values, we constructed microcosms of sterile litter with fungal isolates and incubated them at a range of temperatures and moistures. We collected dry, standing litter from Loma Ridge National Landmark in Southern California (﻿33° 44’ 13.2” N, 117° 42’ 42.0” W, 365 m elevation), which is located on the traditional territory of the Acjachemen and Kizh communities [47, 48]. The study site has an annual mean temperature of 17 °C and means precipitation of 30 cm [49]. Litter predominately consisted of grasses and forbs, including *Avena*, *Bromus*, *Lolium*, *Erodium*, *Lupinus*, and *Stipa pulchra* [50, 51]. We cut, mixed, and ground the litter (~1–2 cm) with coffee grinders. The ground litter was mixed thoroughly to ensure homogeneity before placing 5 g of litter into 120 mL, amber widemouthed jars. The jars and litter were autoclaved for 90 min at 121 °C to sterilize.

We inoculated the jars of sterile litter with one of 15 fungal isolates that were isolated from Loma Ridge in winter 2017. These fungi were identified via Sanger Sequencing using the ITS1F/ITS4 primer sets [52, 53]. The isolates represented 12 different species (some of the isolates were the same species) from both Ascomycota and Basidiomycota phyla (Table S1; Fig. S1). The majority of isolates were Ascomycota, which is representative of the sequences dominating leaf litter from that site [33, 54]. The previously isolated fungi were stored as plugs in sterile water and regrown on potato dextrose agar (with ampicillin and gentamicin) for one week. Afterwards, the fungi were transferred and grown in potato dextrose broth (with ampicillin and gentamicin). After one week of growing in the broth with continuous shaking, we centrifuged and rinsed the hyphae. The hyphal pellet was broken up and diluted with sterile water to an optical density of 0.2 ± 0.02 in order to ensure approximately equal fungal inoculation into each jar [55]. We pipetted 0.5 mL of the dilute fungal hyphae evenly onto the litter in the jars.

After inoculation, we added sterile deionized water to each microcosm so that the percent water holding capacity (WHC) equaled 4, 11, 27, 43, or 50%, and then placed each jar in incubators set at 4, 9, 20, 31, or 36 °C. While some of these moisture × temperature combinations are more likely than others, we chose these levels to capture the range of conditions encountered in the field for these fungi. Field conditions typically vary from hot, dry conditions in the summer to cool, wet conditions in the winter [49]. Microcosms were constructed using a response surface design with 9 moisture and temperature combinations (Table S2), 15 isolates (plus an additional uninoculated control), and 2 sub-replicates for each treatment × isolate combination, for a total of 288 microcosms. We incubated the microcosms (sterile litter, fungi, and water) for five weeks, airing out the jars weekly for at least one minute to avoid anoxic conditions, while maintaining constant moisture levels. The jars were aired out one at a time in a laminar flow cabinet to prevent cross-contamination between samples. After five weeks, we mixed the samples in the jars and froze part of the litter at −20 °C for fungal hyphal biomass measurements and the other part of the litter at −80 °C for extracellular enzyme activity measurements.

### Extracellular enzyme activity and fungal biomass

We measured the potential activities of four extracellular enzymes: cellobiohydrolase (CBH), *β*-glucosidase (BG), *β*-xylosidase (BX), and N-acetyl-*β*-*D*-glucosaminidase (NAG). CBH and BG degrade cellulose, BX degrades hemicellulose, and NAG degrades chitin, which are common components of plant fibers and fungal cell walls that decomposer fungi degrade [56, 57]. Sample homogenates and fluorometric enzyme assays were conducted according to methods described in Alster et al. [43, 58]. In brief, we mixed 0.2 g of litter with 75 ml of 25 mM maleate buffer (pH 6.0) using a Polytron automated homogenizer. We added 200 μl of this homogenate and 50 μl of a fluorescent substrate to a 96-well plate. Each sample was replicated eight times. We used 4-Methylumbelliferone as a standard and included controls for the fluorescence of the homogenate, buffer, and substrates. After a one-hour incubation at room temperature, we added 10 μl of NaOH (1 M) to terminate the assay. The plates were read at 365 nm excitation and 450 nm emission. The final activity was calculated using the standard, controls, and average fluorescence of the eight replicates, adjusting for litter mass.

We measured fungal biomass as an additional metric of plasticity. We also used the fungal biomass measurements to standardize the potential EEA values so that EEA values were not simply a reflection of the change in biomass with moisture and temperature (i.e., enzyme activity may be a function of biomass). To calculate fungal hyphal biomass we used a procedure modified from Allison et al. [59], which involved extracting and staining the fungal hyphae. Frozen litter (0.5 g) was stirred in a 39.5 g/L sodium hexametaphosphate solution and subsamples (5 mL) were vacuum-pumped through a 0.2 µm nylon filter. These filters were stained with acid fuchsin and this procedure was repeated twice per sample. Filters were mounted on slides and dried overnight at 60 °C. We took 5 photos from each filter (10 photos total per sample) using an Axioplan 2 imaging microscope. We measured fungal hyphal length using AxioVision and calculated fungal hyphal length per gram of litter [60]. We converted fungal hyphal length to biomass assuming a fresh density of 1.1 g cm^−3^, 33% dry mass, 40% C in dry mass, and a 5.2 µm hyphal diameter [51, 59, 61–63]. These estimates were based on average hyphal diameter in soil fungi and are representative of the fungal taxa in our study. We divided each EEA value by fungal biomass so that units were µmol h^−1^ µg^−1^ C.

### Statistical analysis

To test Hypotheses 1 and 2, we examined how point values varied across the temperature and moisture gradients, and how plasticity traits varied among fungi. All statistics were conducted in R version 3.5.3 [64]. We used response surface methodology [65] to characterize the topography of EEA and fungal biomass change with temperature and moisture for each isolate. This approach allows us to explore the relationship between multiple explanatory variables (i.e., moisture and temperature) on the response variables (i.e., fungal biomass and EEA) without having to take measurements at every point along each gradient [66, 67]. Although we included two sub-replicates at every measured point, response surface methodology reduces the need for replication [68, 69] because there is hidden replication in the factorial design; the average values and differences between treatments at any given moisture-temperature level can always be estimated. Thus, response surface methodology is ideal for measuring phenotypic plasticity along multiple environmental gradients.

The response surface methodology uses a linear regression model to estimate a first- and second-order approximation to the topography. Hypothesis tests can then be used to determine which topographic approximation is best for each isolate, and which isolates have different approximate surfaces. The regression model terms are grouped into three categories: first-order, two-way interaction, and pure-quadratic. The first term is all that is needed for a first-order approximation, and if either of the second two terms are included then a second-order approximation is needed. We determined which terms were necessary by backward selection using sequential analysis of variance (ANOVA). First, the two-way interaction is tested, then the pure-quadratic, and finally the first-order terms are tested. This step is important to determine the approximate relationship between the independent variables (i.e., temperature and moisture) and the dependent variable (i.e., each trait) [65] and ensures an appropriate relationship between points (linear or non-linear). Additionally, we added isolate-specific intercepts and included interaction terms with isolate to allow for isolate-specific effects and to test for differences in response surfaces among isolates. We found that potential activities of CBH, BG, BX, and NAG all were significant for the pure-quadratic interaction (Table S3), but not for the two-way interaction (*n* = 270). Thus, we fit CBH, BG, BX, and NAG according to Eq. (1):

1 $$Y_{ i} = \beta _{ i} + \beta _{ ti}X_{ t} + \beta _{ mi}X_{ m} + \beta _{ ti2}X^{2}_{ t} + \beta _{ mi2}X^{2}_{ m},$$

where $Y_{\it{i}}$ represents the vector of logged values of CBH, BG, BX, or NAG for isolate *i*, $\beta _{\it{i}}$ is the isolate’s intercept, $\beta _{\it{ti}}$ is the isolate-specific regression coefficient for temperature, $\beta _{\it{mi}}$ is the isolate-specific regression coefficient for moisture, $X_{\it{t}}$ is the temperature (°C), and $X_{\it{m}}$ is moisture (%WHC). For fungal biomass, only the first-order term was significant (Table S3). Thus, we fit fungal biomass according to Eq. (2):

2 $$Y = \beta _{ i} + \beta _{ ti}X_{ t} + \beta _{ mi}X_{ m}$$

Using these optimized models, we ran a regression analysis and ANOVA to evaluate how temperature and moisture interacted with each isolate to determine significant differences in plasticity traits (*n* = 270). The R^2^ for these model fits ranged from 0.53–0.97 (*P* < 0.0001). In addition, we ran another ANOVA to determine the significance of temperature, moisture, and fungal isolate type on point values across all fungal isolates (*n* = 270). We used the log-transformed values for these analyses to improve normality after inspection of residual plots for un-logged trait values. Uncertainty is accounted for in the model by using the normalized variances as weights.

To test for where significant differences occurred at each specific point value on the response surface, we created an array of points representative of the moisture and temperature levels we measured (330 total moisture and temperature points). We then used the ‘emmeans’ package to compute the pairwise estimated marginal means based on the ANOVA and contrasts between the specific points for each isolate [70]. This generated a *P*-value statistic and size difference for every point in the array. We were then able to determine if there was a significant difference, and the magnitude of the difference, between the same points along the response surface for pairs of isolates. To visualize the data, we also created contour plots for each isolate using response surface regression generated from the ‘rsm’ package [65]. Significant effects of temperature and moisture on point values would support Hypothesis 1. Significant interactions between isolates and temperature (or between isolates and moisture) would indicate that plasticity traits differ across fungal isolates, in support of Hypothesis 2.

To test Hypothesis 3 and determine if point values were more similar for more closely related fungi, we conducted phylogenetic signal tests. For each point measured in the response surface, we calculated Pagel’s *λ* [71] and Bloomberg’s K [72] test statistics for each trait using the ‘phytools’ package [73]. These tests quantify if traits from more closely related species are most similar. We used the mean value for each fungal species at each moisture × temperature point for the phylogenetic signal tests (*n* = 12). For species with multiple isolates measured, the mean values at each moisture × temperature point were also averaged. We inferred the presence of a phylogenetic signal if both Pagel’s *λ* and Blomberg’s K tests resulted in *P* < 0.05. Bonferroni adjusted *P*-values were also calculated for reference to account for multiple comparisons. The presence of a significant phylogenetic signal would support Hypothesis 3.

## Results

**Fig. 2:**
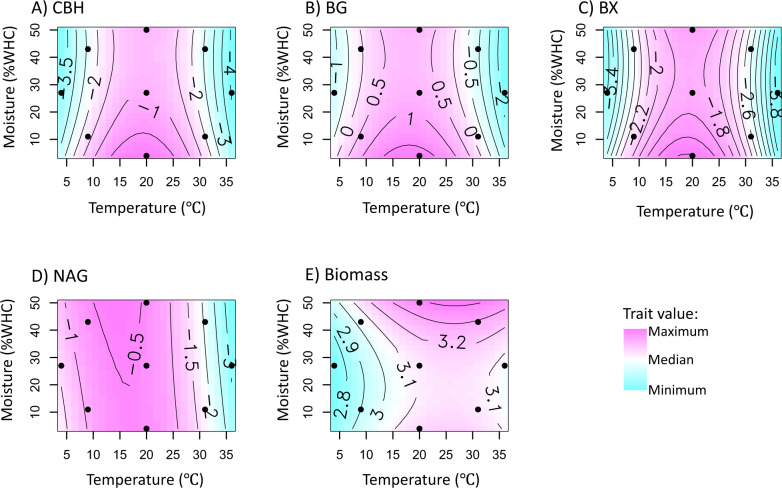
Response surface plots. Contour plots of log-transformed data for the average activities of all of the isolates for (**A**) cellobiohydrolase (CBH), (**B**) *β*-glucosidase (BG), (**C**) *β*-xylosidase (BX), and (**D**) N-acetyl-*β*-*D*-glucosaminidase (NAG), and (**E**) fungal biomass across the moisture-temperature gradient (*n* = 18 for each response surface). Each response surface is on a separate scale, as indicated by the numbers on the lines on each contour plot. All maximum values are pink, and all minimum values are blue. The small black circles indicate incubation conditions. Response surfaces for each fungal isolate individually can be found in Figures S2–S6.

Point values varied significantly with temperature for BG, NAG, and fungal biomass, and with moisture for CBH and BG, which supported Hypothesis 1 (Table S4). For CBH, BG, and BX, point values peaked at moderate temperatures and lower moisture (Fig. 2 and Figs. S2–S4). For NAG, point values tended to decline with temperature (Fig. 2 and Fig. S5). In contrast, on average fungal biomass increased with temperature and did not vary significantly with moisture (Fig. 2 and Fig. S6).

Interactions between isolates and temperature were significant for CBH, BG, BX, NAG, and fungal biomass (Table 1), indicating that fungal isolates differed in their plasticity traits with respect to temperature. However, interactions between isolates and moisture were only significant for BG (Table 1). Therefore, Hypothesis 2 was partially supported. These findings are visualized in Figs. S7–S11 (Tables S5–S9) where differences between points along the response surface are most evident at the temperature extremes, such as higher than 30 °C.

**Table 1:** ANOVA results for cellobiohydrolase (CBH), $\beta$-glucosidase (BG), $\beta$-xylosidase (BX), N-acetyl-$\beta$-$D$-glucosaminidase (NAG), and fungal biomass demonstrating the effect of the isolate as well as temperature and moisture within response surfaces (*n* = 270).

		CBH		BG		BX		NAG		Fungal biomass	
	df	F	*P*	F	*P*	F	*P*	F	*P*	F	*P*
Isolate	15	21.770	**<0.0001**	1.506	0.106	33.264	**<0.0001**	12.013	**<0.0001**	409.314	**<0.0001**
Isolate*Temperature	15	1.271	0.224	2.743	**0.001**	1.207	0.269	2.618	**0.001**	2.364	**0.004**
Isolate*Moisture	15	1.411	0.145	2.298	**0.005**	0.768	0.712	1.232	0.250	1.671	0.058
Isolate*(Temperature^2)	15	6.940	**<0.0001**	8.003	**<0.0001**	4.141	**<0.0001**	2.900	**<0.0001**	NA	NA
Isolate*(Moisture^2)	15	0.212	0.999	0.306	0.994	0.269	0.997	0.355	0.988	NA	NA

*Because higher-order interactions were not significant for fungal biomass (Table S3), there are no results for the quadratic terms. Bold indicates significance (*P* < 0.05).

**Fig. 3:**
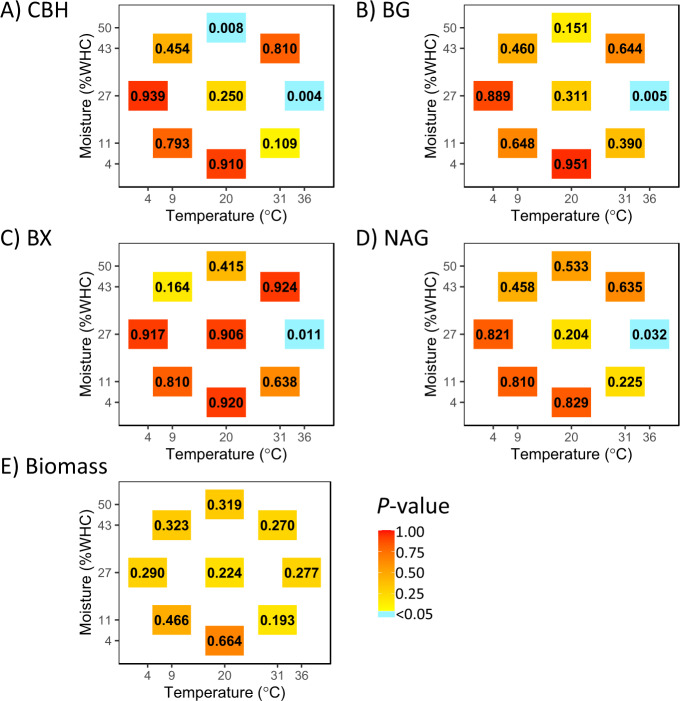
Heat map representing the strength of the phylogenetic signal. (**A**) cellobiohydrolase (CBH), (**B**) *β*-glucosidase (BG), (**C**) *β*-xylosidase (BX), and (**D**) N-acetyl-*β*-*D*-glucosaminidase (NAG), and (**E**) fungal biomass across the moisture-temperature gradient (*n* = 12 for each *P*-value). The color gradient signifies the *P*-value (unadjusted) associated with Blomberg’s K test at each specific point on the moisture-temperature gradient, with blue values indicating a significant phylogenetic signal (*P* < 0.05). The numbers in each colored box represent the *P*-value at that specific moisture × temperature combination.

All of the EEAs displayed phylogenetic signals at the highest temperature point (36 °C, 27% WHC), although with the multiple comparison adjustment, some of these relationships were only marginally significant (Fig. 3; Table S2). A phylogenetic signal was also found at the highest moisture level (20 °C, 50% WHC) for CBH activity, however this relationship was no longer significant when accounting for multiple comparisons. No phylogenetic signal for point values at any other moisture or temperature levels was observed. Fungal biomass also did not have a phylogenetically signal at any point along the temperature and moisture gradients for the isolates measured here. Overall, Hypothesis 3 was also partially supported. Of related interest, phenotypic plasticity for isolates of the same species was sometimes not consistent. For example, in Fig. S6, the biomass of *Coprinellus aff xanthotrhis* is not identical across all three strains.

We summarize our findings with an example illustrated in Fig. 1B. Response surfaces for NAG activity for three isolates, *Trichoderma koningii* (phylum Ascomycota), *Sarocladium implicatum* (phylum Ascomycota), and *Coprinellus aff xanthotrhis* (phylum Basidiomycota), are shown. For all three isolates, point values vary across the temperature gradient, and to a lesser degree the moisture gradient (Hypothesis 1). Plasticity traits were also different between the three isolates (Hypothesis 2). Although NAG activity peaked at moderate temperature and moisture in all isolates, point values diverge at high and low temperatures, so that the two Ascomycota species were more similar to one another than to the Basidiomycota species (Hypothesis 3).

## Discussion

In this study, we examined the phenotypic plasticity of EEA and fungal biomass and the degree to which this phenotypic plasticity has a phylogenetically signal. We found that plasticity traits varied for fungi (Fig. 2), mainly due to divergence in trait values at environmental extremes, such as at temperatures greater than 30 °C (Fig. S7–S11). Additionally, at the highest temperature, closely related fungi tended to display more similar EEAs than did more distantly related fungi (Fig. 3). Likewise, at the highest moisture level, CBH activity had a phylogenetic signal, although this signal was not significant when adjusting for multiple comparisons (Fig. 3). In other words, fungal contributions to organic matter breakdown under extreme conditions tended to be more phylogenetically related than activities under moderate conditions.

Differentiation of fungal responses under more extreme temperatures (Fig. S7–S11) suggests that with global warming, fungal biodiversity may increasingly influence organic matter dynamics. Biodiversity has been a major focus in trait-based microbial studies [74, 75]. In the current study, fungal isolates did not differ markedly in EEA point values when exposed to moderate conditions. While we only examined 12 fungal species in this study, this finding suggests that changes in fungal biodiversity at moderate temperatures and moistures might not have a substantial effect on organic matter breakdown in this southern California grassland. However, because EEA point values varied more strongly at the warmer temperatures, biodiversity may more strongly influence ecosystem function as the climate warms. Under these higher temperatures, some fungi may be less attuned for optimal growth and survival, leading to greater differentiation in phenotypic plasticity between fungal strains. This hypothesis is supported by other studies noting the importance of diversity in maintaining ecosystem function with warming [76–78]. Likewise, under ambient conditions, functional differences between soil microbial communities from a grassland ecosystem were limited, but under heat stress, biodiversity mattered [79].

The appearance of a phylogenetic signal for EEA at the most extreme environmental conditions is perhaps surprising. We may expect to find a stronger phylogenetic signal under milder environments because of increased competition between species [80, 81]. However, differences in EEA point values at environmental extremes may have resulted from a trade-off between enzyme production and expression of other traits more directly related to stress tolerance [82, 83]. For example, under high stress, fungi might invest resources toward the production of stress proteins rather than degradative enzymes [84]. Perhaps certain fungi have developed different types of adaptations for increasing and decreasing extracellular enzyme production under these more stressed environmental conditions [85–87]. It is possible that measurement of other traits, such as peroxidase or phenol oxidase enzyme activities, may have resulted in different types of response surfaces and phylogenetic relationships than what we observed here. Additionally, perhaps the seasonality of when the fungi were isolated could have influenced the observed results. Because the fungi were isolated from litter collected in cooler months, that may explain the greater similarity between trait responses at the cooler and drier temperatures.

In contrast to the observed phylogenetic signals at certain points along the response surface, we also found some evidence that isolates of the same species differ in their plasticity (e.g., in Figure S6 plasticity differed for fungal biomass between the three stains of *Coprinellus aff xanthotrhis*). Perhaps these strains were isolated from different microclimates in the soil/litter. Thus, despite being the same species, adaptations to different microclimate conditions may have resulted in differences in plasticity. Alternatively, experimental errors, such as unintended anoxic conditions in some of the jars, could have contributed to this unexpected variation. It would be useful to investigate this intraspecies variation in phenotypic plasticity in future studies to elucidate any mechanisms at play.

These results also demonstrate the value of measuring traits under more than one environmental condition. In the current study, point values of biomass and EEA varied most with temperature (Table 1), which is consistent with assertions that measurements at multiple growth temperatures could be particularly informative [44, 88, 89]. Temperature can shape enzyme production and activity through structural adaptations of isoenzymes and through temperature sensitivity of enzyme kinetics [86, 90]. Potential BG activity varied with moisture as well (Fig. 2). While we may have expected greater differentiation of biomass and EEA point values between species under less common environmental conditions (e.g., very wet conditions), we did not find evidence of this except for with BG activity. Moisture regulates enzyme production and activity, in part by altering diffusion rates [91]. Perhaps the BG enzyme-substrate complex is relatively sensitive to changes in diffusion rates, compared with the other EEA measured here, leading to a greater need for BG production under dry conditions to offset lower diffusion.

In conclusion, our work highlights the importance of examining phenotypic plasticity as a fungal trait. We found that plasticity traits differed among isolates, with trait values varying most at environmental extremes. This work suggests that fungal biodiversity may become increasingly important in maintaining ecosystem function with global warming, in this southern California ecosystem, since fungal responses tended to diverge at higher temperatures. While this work provides a starting point, we would encourage other scientists to use this response surface approach to investigate the phenotypic plasticity of traits from other microbes. Overall, understanding the phenotypic plasticity of fungal traits across environmental gradients may be useful in better connecting microbial trait response to ecosystem function and biogeochemical cycling.

## Supplementary information

Supplementary figures

Table S1

Table S2

Table S3

Table S4

Table S5

Table S6

Table S7

Table S8

Table S9
