# Supplementary material for: Phenotypic plasticity of fungal traits in response to moisture and temperature
Source: ISME Commun. 2021 Aug 28;1:43. doi: 10.1038/s43705-021-00045-9 (PMC9723763; doi:10.1038/s43705-021-00045-9)
Supplement: Supplementary file 1 — Supplementary figures [file 43705_2021_45_MOESM1_ESM.pdf]

Supplemental Figures:

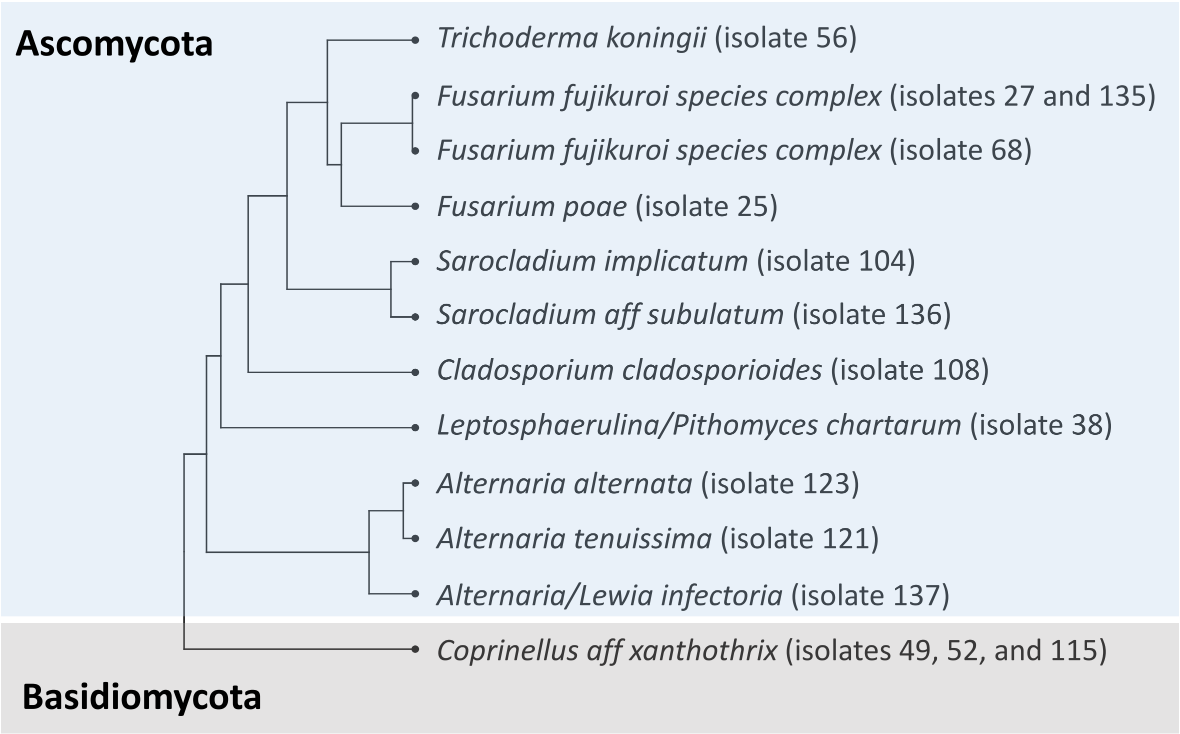

Figure S1. Phylogenetic tree of the fungal isolates used in this experiment.

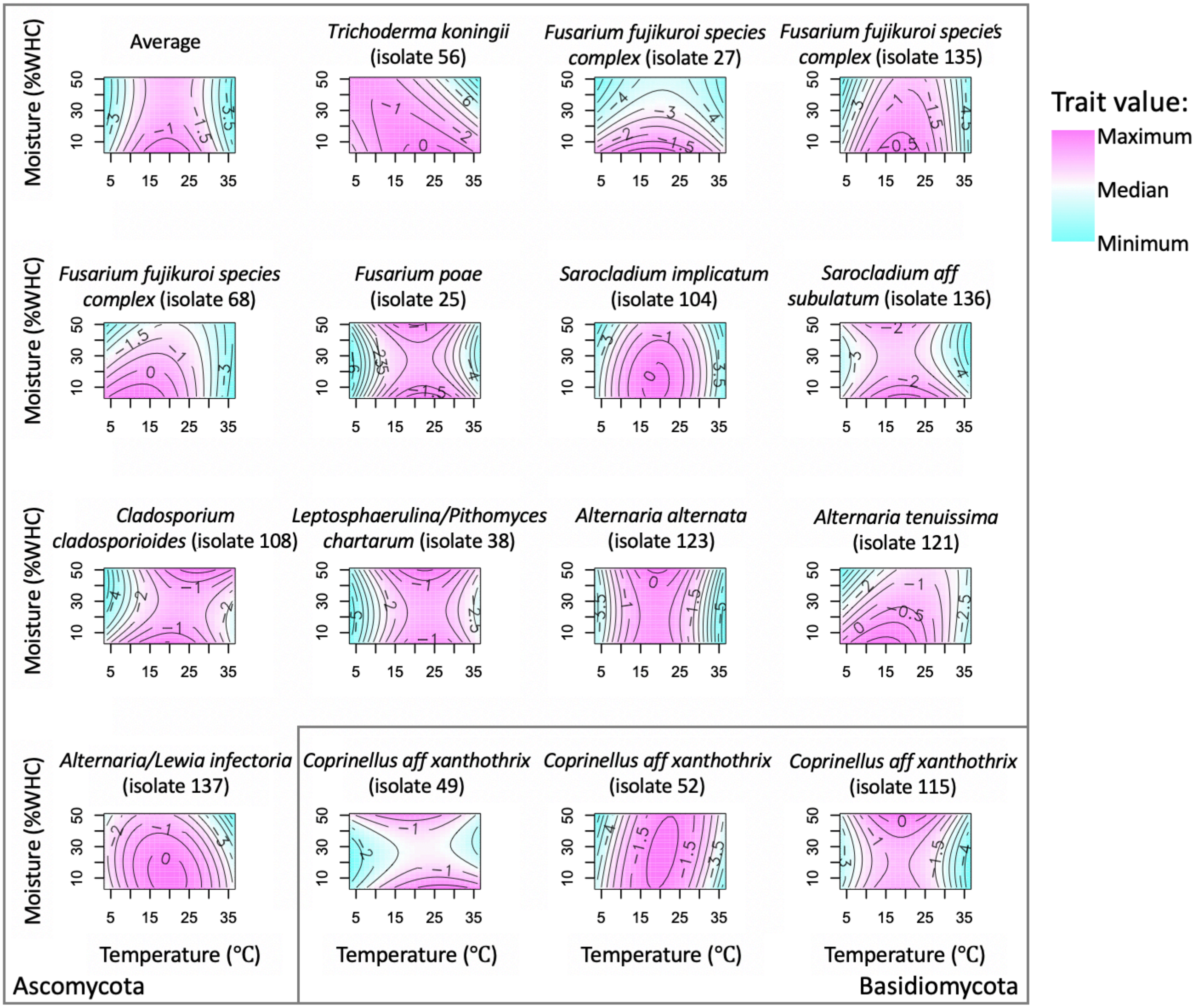

Figure S2. Contour plots of log-transformed data for cellobiohydrolase. Each plot represents values for an isolate (or the average of all isolates) along the moisture and temperature gradient (n = 18 for each response surface). Each response surface is on a separate scale, as indicated by the numbers on the lines on each contour plot. Regardless, all maximum values are pink, and all minimum values are blue. Additional information regarding each fungal isolate can be found in Table S1.

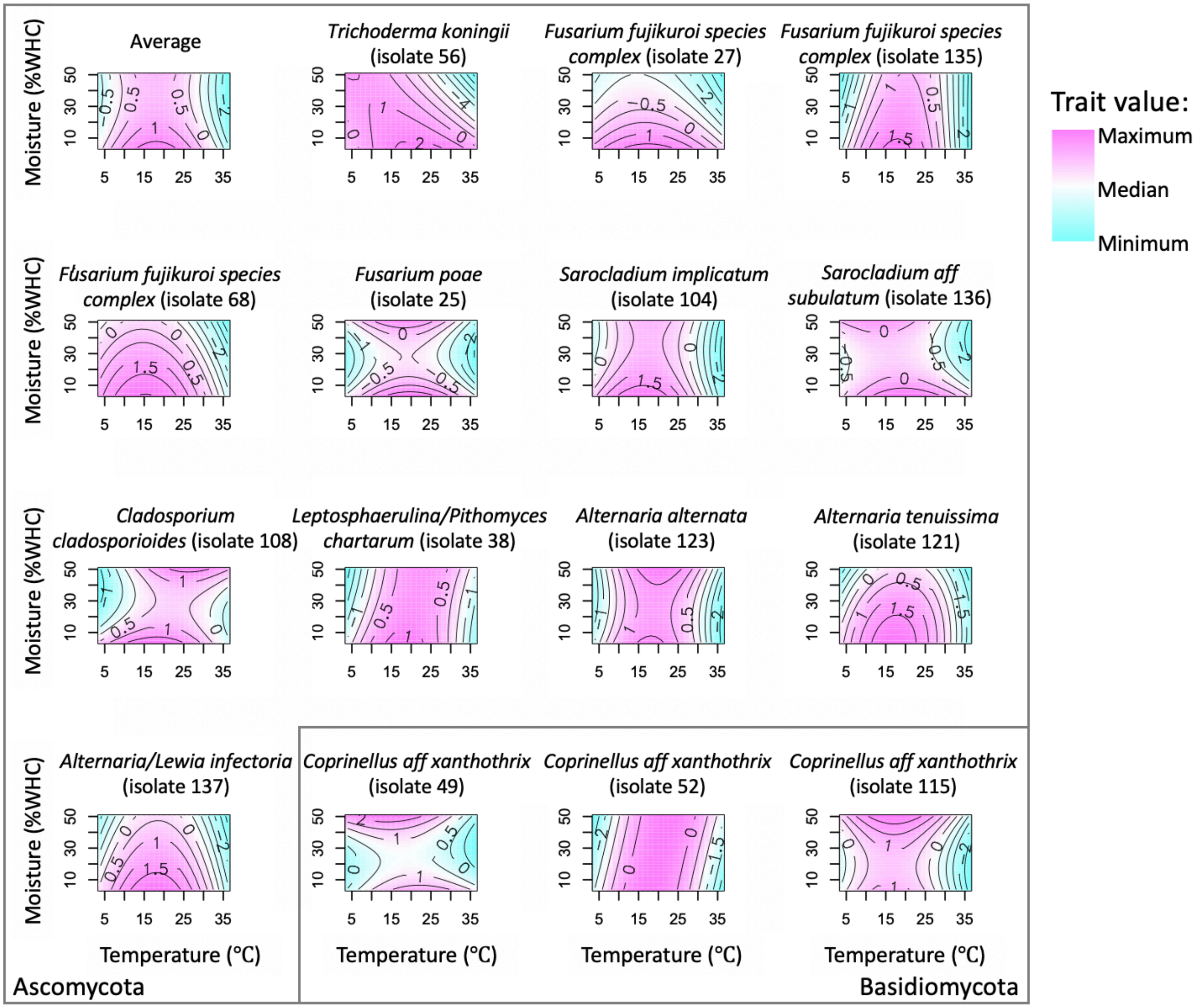

Figure S3. Contour plots of log-transformed data for β-glucosidase. Each plot represents values for an isolate (or the average of all isolates) along the moisture and temperature gradient (n = 18 for each response surface). Each response surface is on a separate scale, as indicated by the numbers on the lines on each contour plot. Regardless, all maximum values are pink, and all minimum values are blue. Additional information regarding each fungal isolate can be found in Table S1.

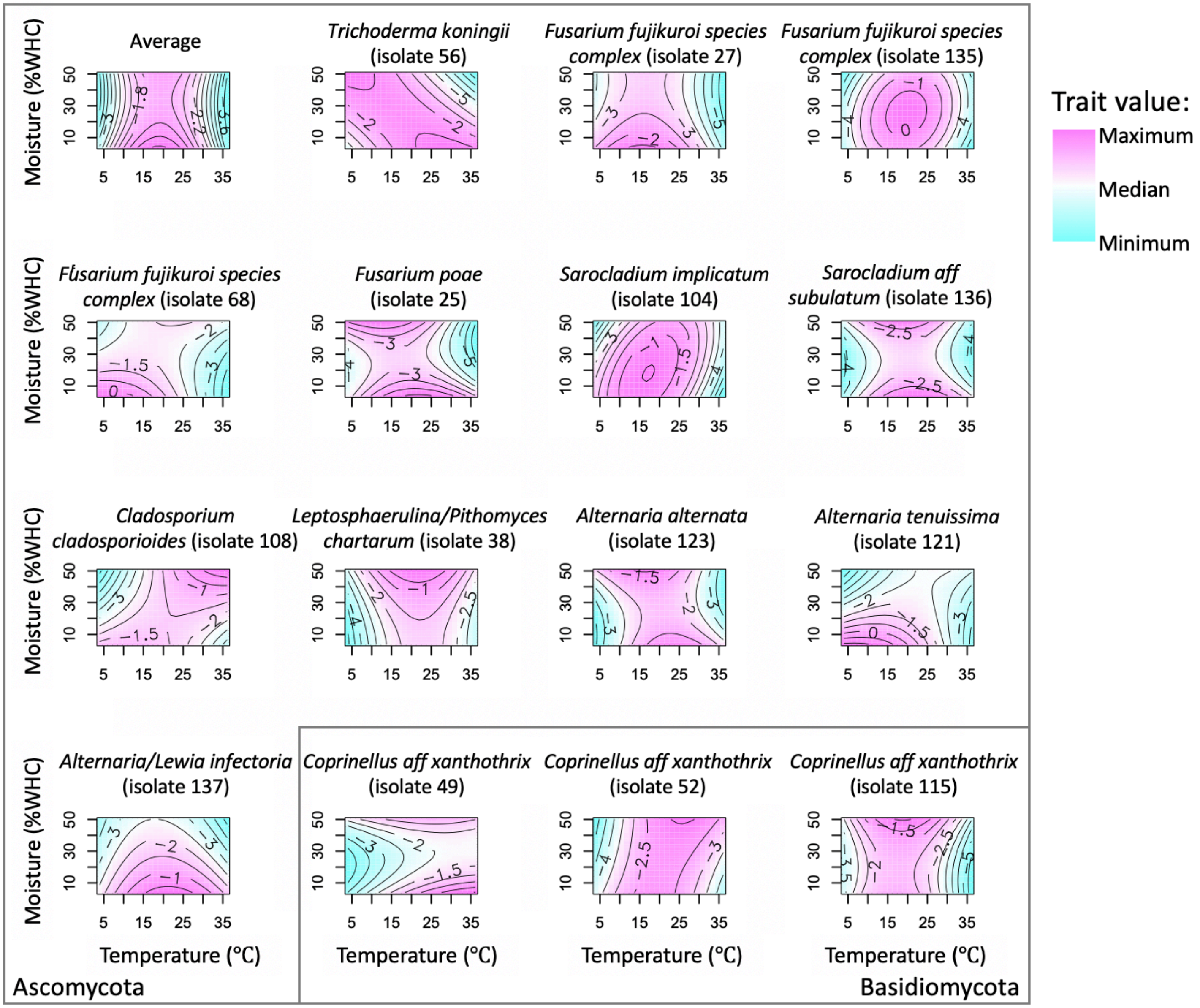

Figure S4. Contour plots of log-transformed data for β-xylosidase. Each plot represents values for an isolate (or the average of all isolates) along the moisture and temperature gradient (n = 18 for each response surface). Each response surface is on a separate scale, as indicated by the numbers on the lines on each contour plot. Regardless, all maximum values are pink, and all minimum values are blue. Additional information regarding each fungal isolate can be found in Table S1.

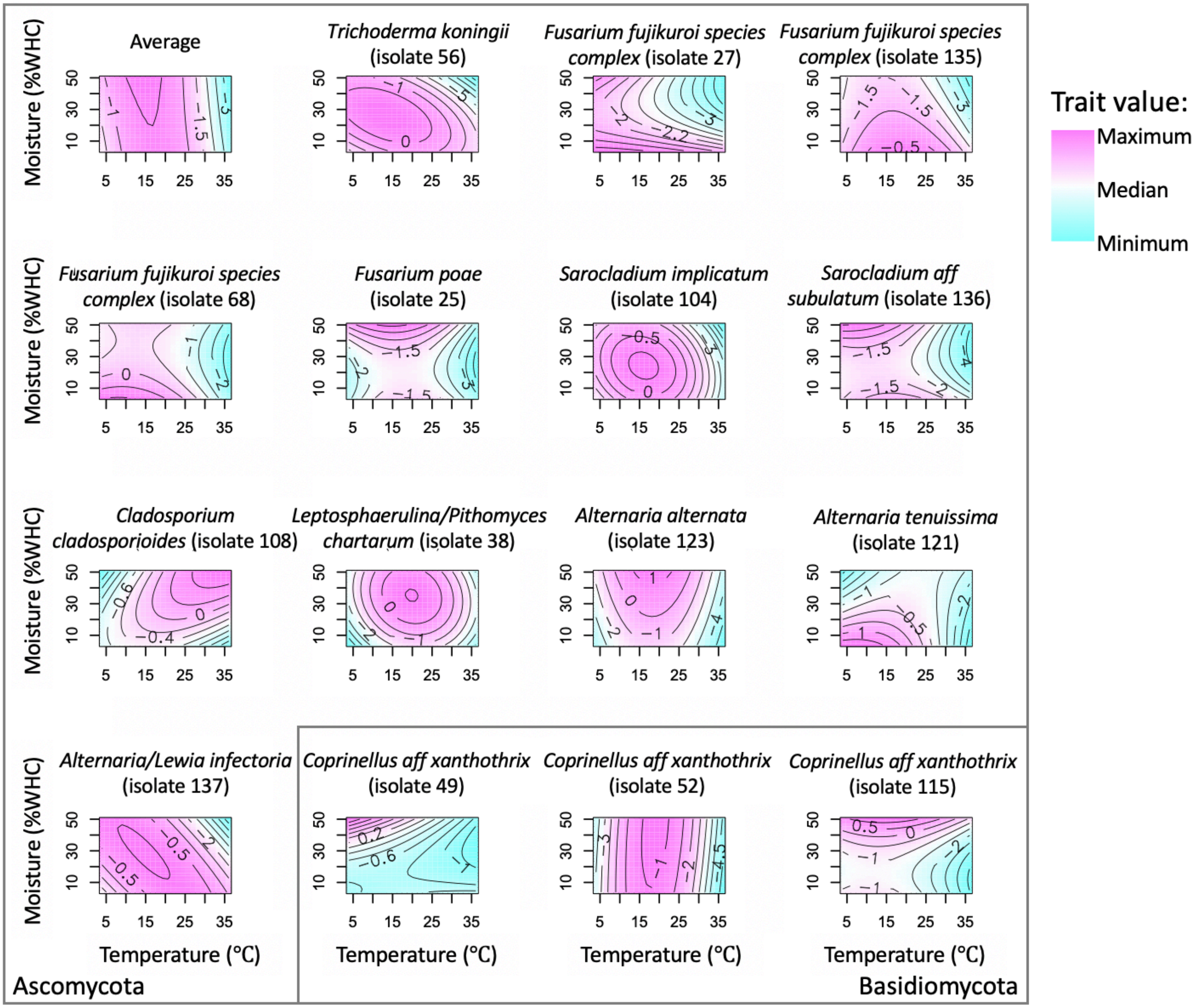

Figure S5. Contour plots of log-transformed data for N-acetyl-β-D-glucosaminidase. Each plot represents values for an isolate (or the average of all isolates) along the moisture and temperature gradient (n = 18 for each response surface). Each response surface is on a separate scale, as indicated by the numbers on the lines on each contour plot. Regardless, all maximum values are pink, and all minimum values are blue. Additional information regarding each fungal isolate can be found in Table S1.

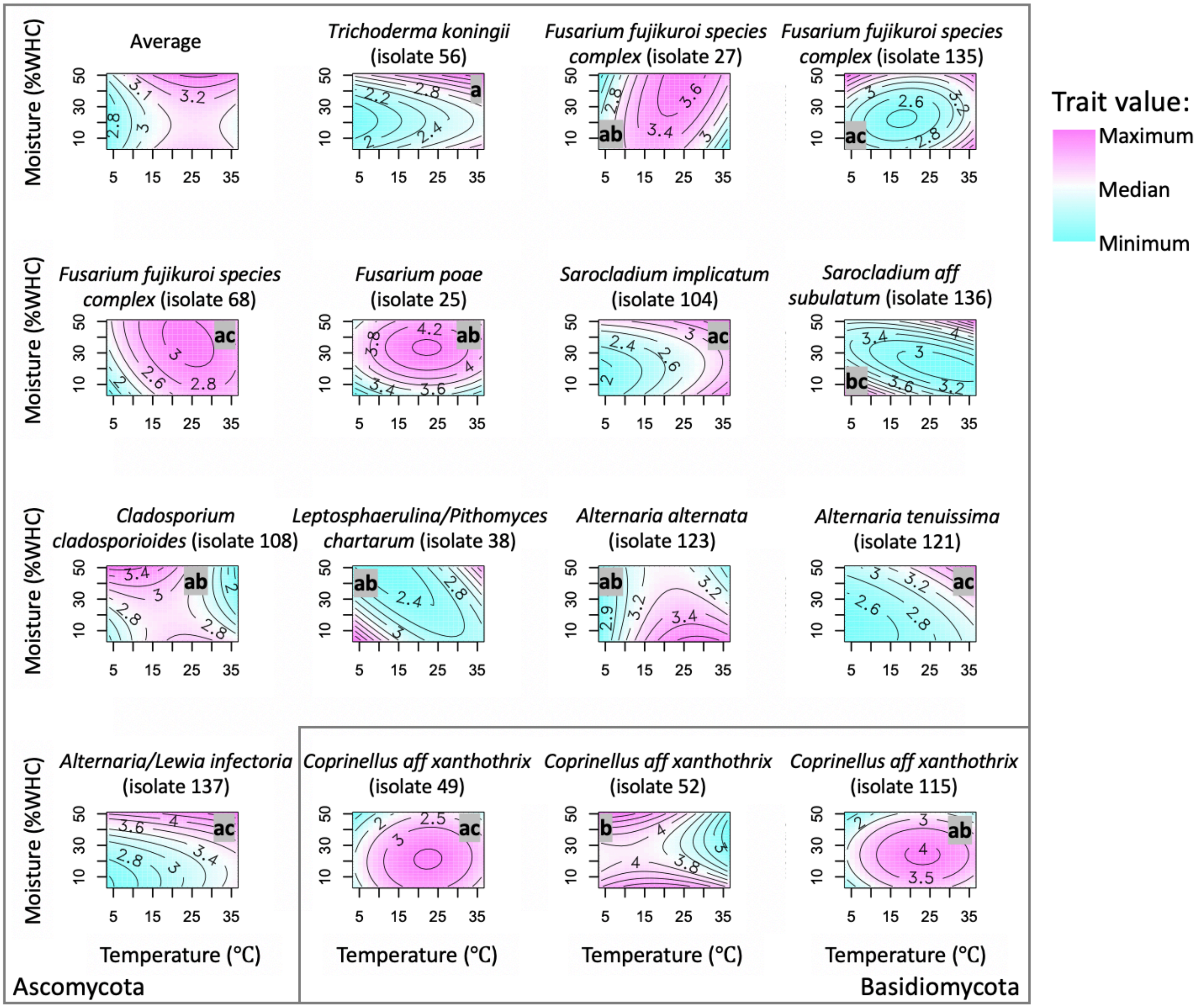

Figure S6. Contour plots of log-transformed data for fungal biomass. Each plot represents values for an isolate (or the average of all isolates) along the moisture and temperature gradient (n = 18 for each response surface). Each response surface is on a separate scale, as indicated by the numbers on the lines on each contour plot. Regardless, all maximum values are pink, and all minimum values are blue. For fungal biomass, isolate type was significant in explaining variation in the point values (Table S4); the letters indicated significant differences in these values ($P < 0.05$). Additional information regarding each fungal isolate can be found in Table S1.

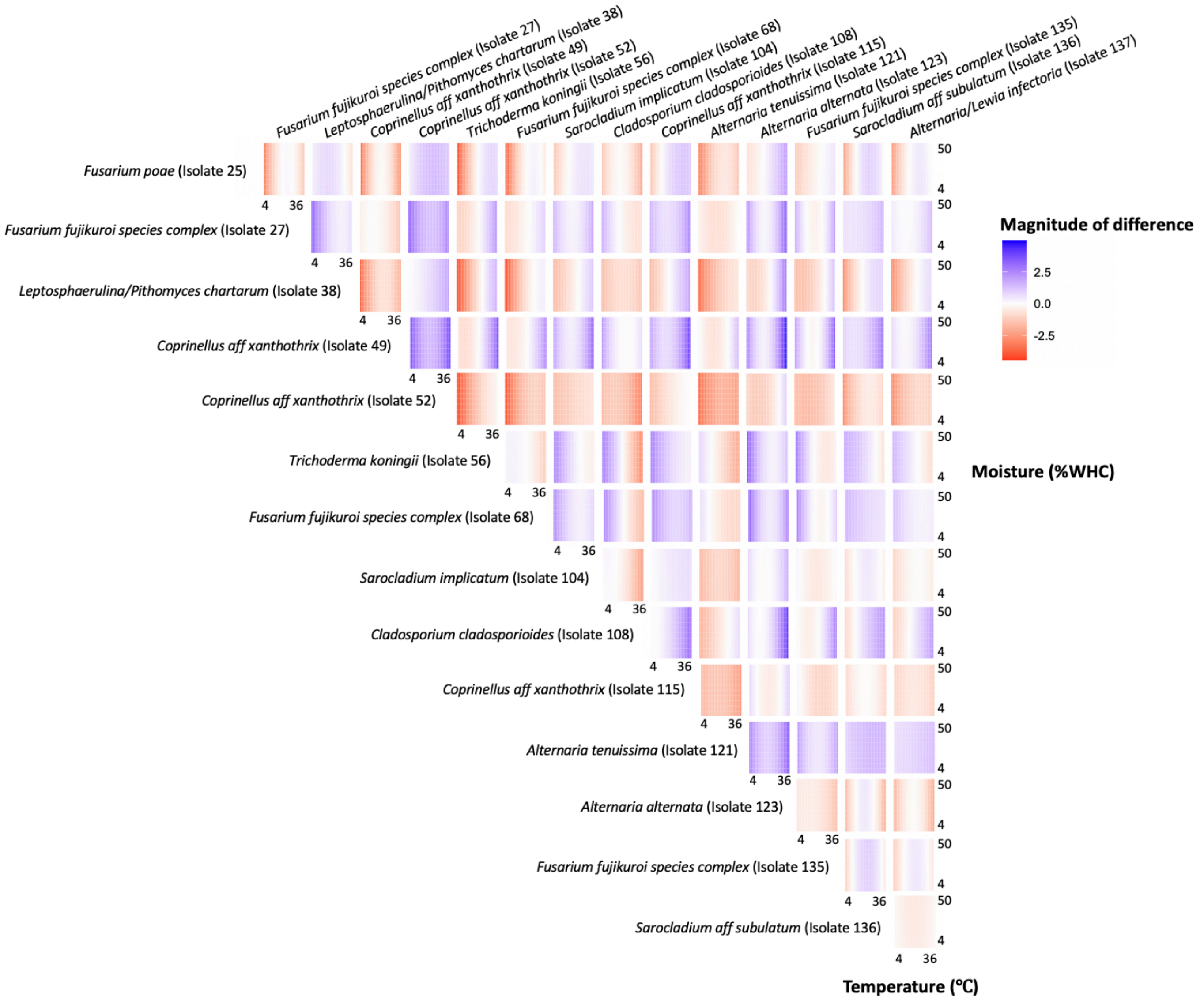

Figure S7. Magnitude of difference between pairs of isolates (isolate in the row minus isolate in the column) at each point across the response surface for cellobiohydrolase (n = 330 for each plot, based on estimates from n = 18 measured point values). The blue color indicates a positive difference and the red color indicates a negative difference between the trait pairs. Differences are non-significant (*P* > 0.05). Information regarding each fungal isolate can be found in Table S1 and pairwise differences between points can be found in Table S5.

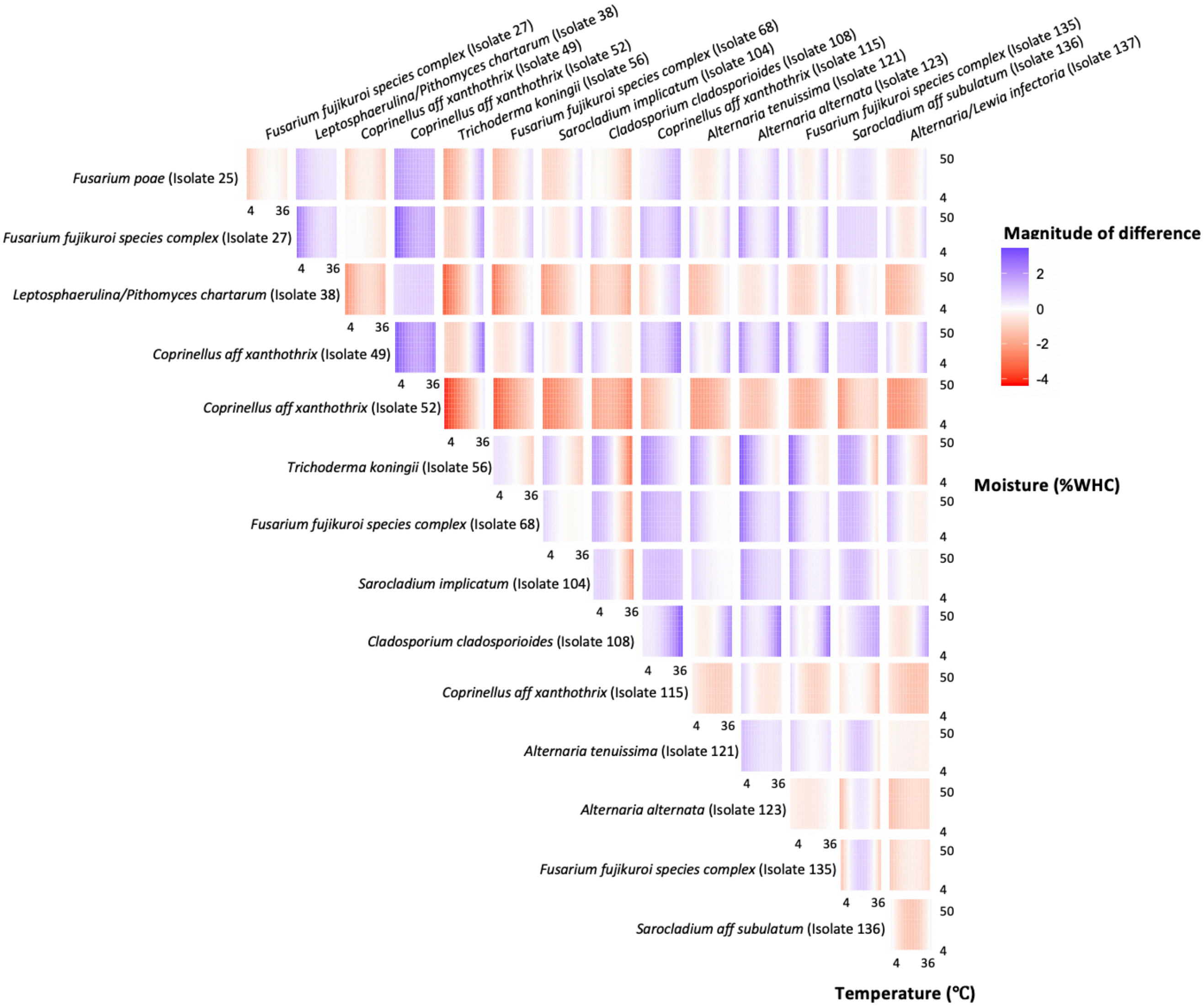

Figure S8. Magnitude of difference between pairs of isolates (isolate in the row minus isolate in the column) at each point across the response surface for β-glucosidase (n = 330 for each plot, based on estimates from n = 18 measured point values). The blue color indicates a positive difference and the red color indicates a negative difference between the trait pairs. Differences are non-significant ($P > 0.05$). Information regarding each fungal isolate can be found in Table S1 and pairwise differences between points can be found in Table S6.

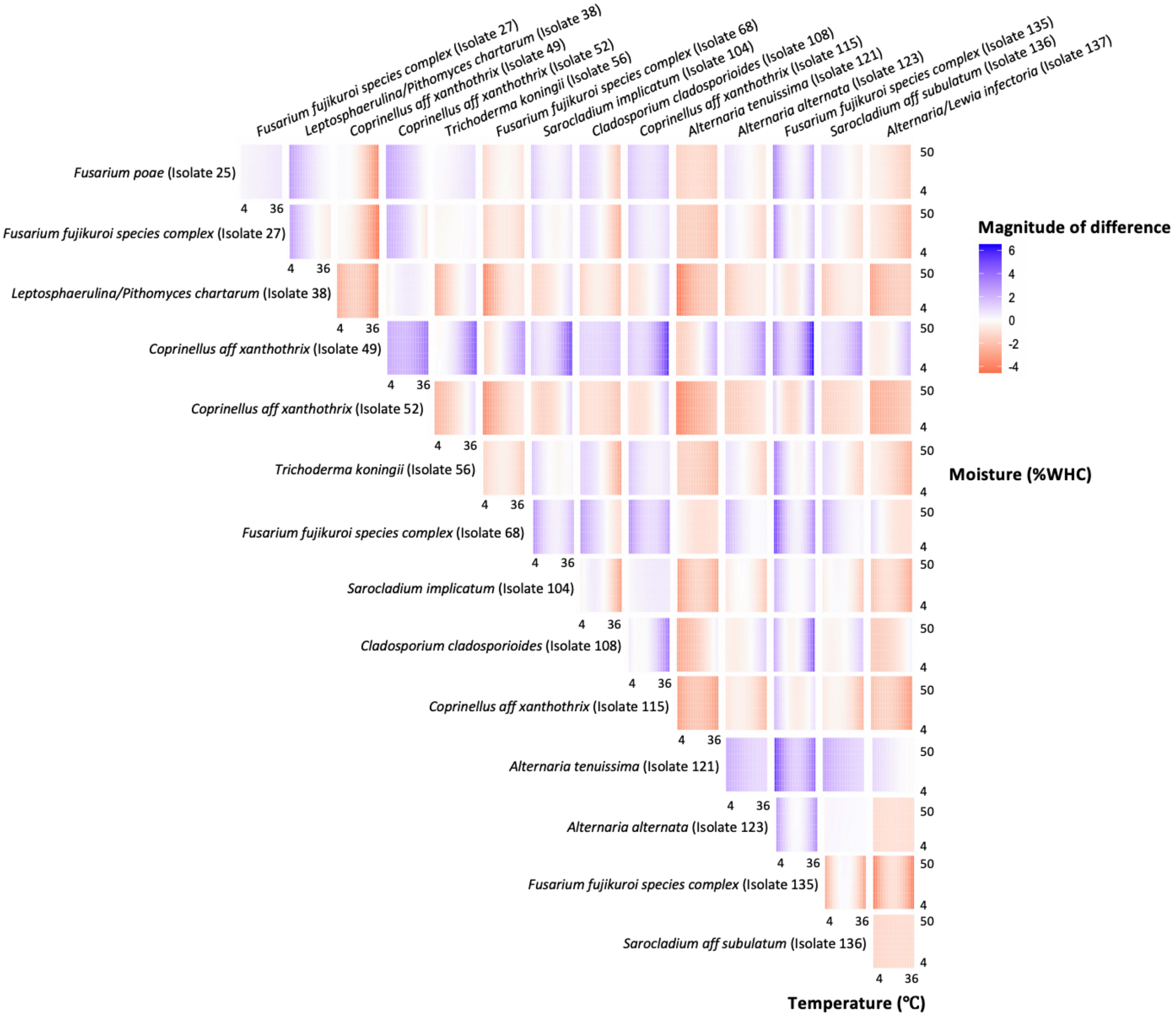

Figure S9. Magnitude of difference between pairs of isolates (isolate in the row minus isolate in the column) at each point across the response surface for β-xylosidase (n = 330 for each plot, based on estimates from n = 18 measured point values). The blue color indicates a positive difference and the red color indicates a negative difference between the trait pairs. Differences are non-significant ($P > 0.05$). Information regarding each fungal isolate can be found in Table S1 and pairwise differences between points can be found in Table S7.

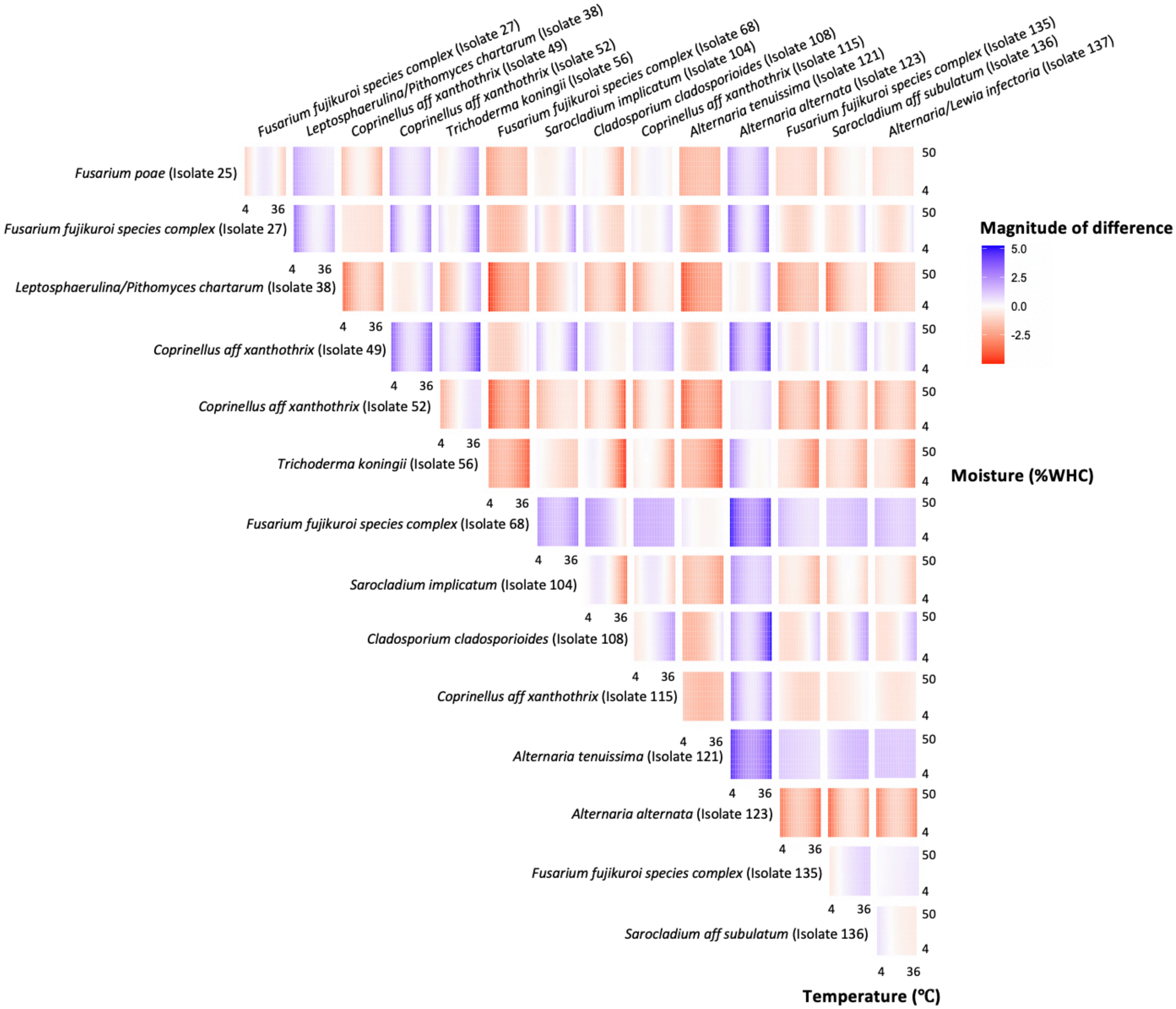

Figure S10. Magnitude of difference between pairs of isolates (isolate in the row minus isolate in the column) at each point across the response surface for N-acetyl-β-D-glucosaminidase (n = 330 for each plot, based on estimates from n = 18 measured point values). The blue color indicates a positive difference and the red color indicates a negative difference between the trait pairs. Differences are non-significant ($P > 0.05$). Information regarding each fungal isolate can be found in Table S1and pairwise differences between points can be found in Table S8.

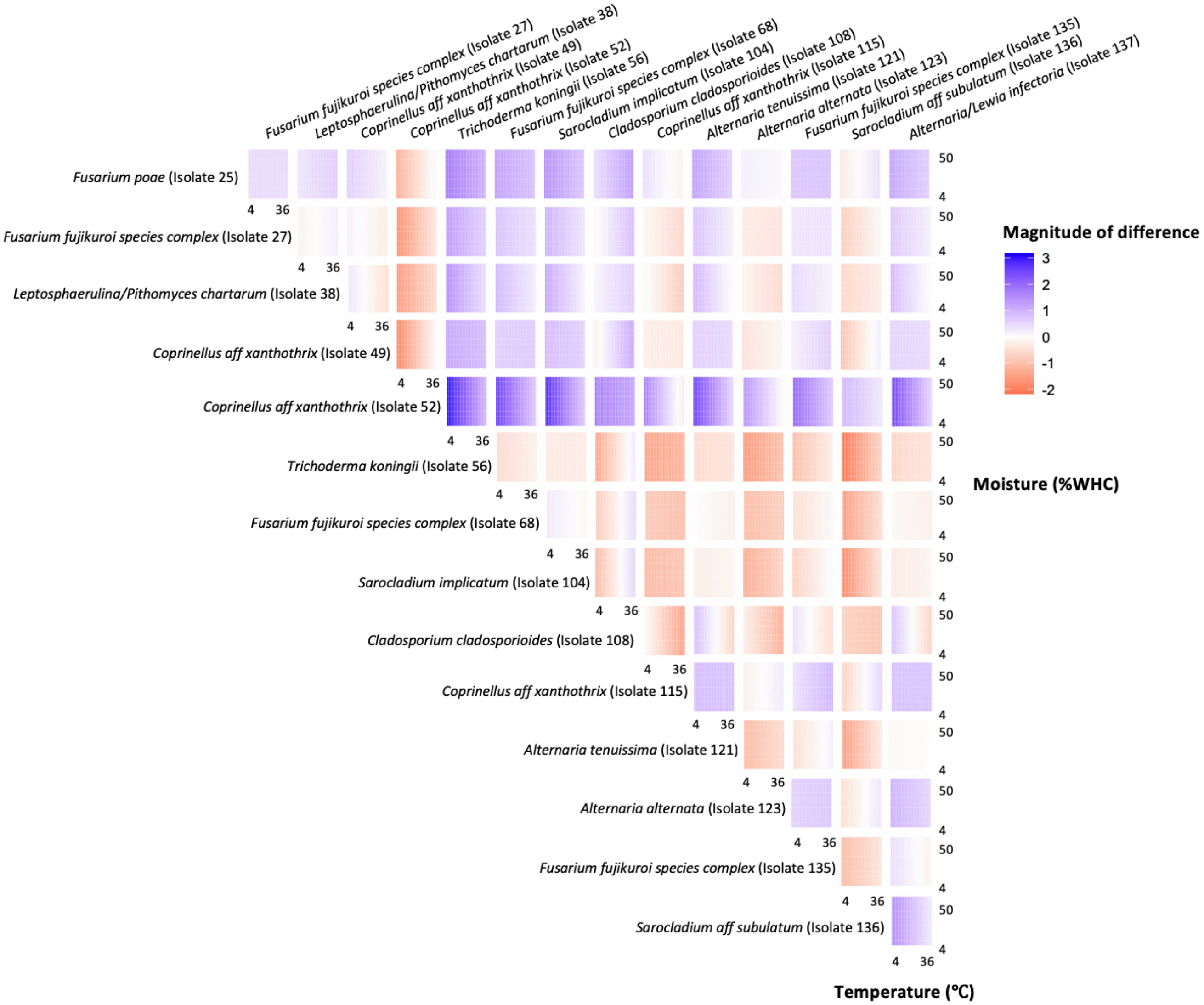

Figure S11. Magnitude of difference between pairs of isolates (isolate in the row minus isolate in the column) at each point across the response surface for fungal biomass (n = 330 for each plot, based on estimates from n = 18 measured point values). The blue color indicates a positive difference and the red color indicates a negative difference between the trait pairs. Differences are non-significant ($P > 0.05$). Information regarding each fungal isolate can be found in Table S1 and pairwise differences between points can be found in Table S9.
